# A New Method for Flow Rate Measurement in Millimeter-Scale Pipes

**DOI:** 10.3390/s130201563

**Published:** 2013-01-25

**Authors:** Haifeng Ji, Xuemin Gao, Baoliang Wang, Zhiyao Huang, Haiqing Li

**Affiliations:** State Key Laboratory of Industrial Control Technology, Department of Control Science and Engineering, Zhejiang University, Hangzhou 310027, China; E-Mails: gaoxuemin1103@163.com (X.G.); blwang@iipc.zju.edu.cn (B.W.); hqli@iipc.zju.edu.cn (H.L.)

**Keywords:** flow rate measurement, Capacitively Coupled Contactless Conductivity Detection, cross correlation flow measurement, millimeter-scale pipes

## Abstract

Combining the Capacitively Coupled Contactless Conductivity Detection (C^4^D) technique and the principle of cross correlation flow measurement, a new method for flow rate measurement in millimeter-scale pipes was proposed. The research work included two parts. First, a new five-electrode C^4^D sensor was developed. Second, with two conductivity signals obtained by the developed sensor, the flow rate measurement was implemented by using the principle of cross correlation flow measurement. The experimental results showed that the proposed flow rate measurement method was effective, the developed five-electrode C^4^D sensor was successful, and the measurement accuracy was satisfactory. In five millimeter-scale pipes with different inner diameters of 0.5, 0.8, 1.8, 3.0 and 3.9 mm respectively, the maximum relative difference of the flow rate measurement between the reference flow rate and the measured flow rate was less than 5%.

## 1. Introduction

Flow rate is one of the most important process parameters in many sectors, such as the chemical, pharmaceutical, petroleum, energy, and power engineering industries, *etc.* [1–5]. Currently, with the development of new technologies, the industrial devices and equipment present a trend of miniaturization [6–9]. More and more millimeter-scale devices/equipment are appearing, such as tube reactors, tube heat exchangers, *etc.* [6–8]. There is a practical need of effective techniques to measure the flow rate in millimeter-scale pipes.

Some commercial flowmeters may be used in millimeter-scale pipes, such as the constriction flowmeter, rotameter, turbine flowmeter, electromagnetic flowmeter, coriollis flowmeter, *etc.* [3–5,10,11]. The constriction flowmeter, rotameter and turbine flowmeter have constriction elements or moving parts in the pipe that disturb the fluid flow and cause a pressure drop. The Coriollis flowmeter also disturbs the fluid flow. Furthermore, this kind of flowmeter has the disadvantage of high cost. Although the electromagnetic flowmeter do not disturb the fluid flow, electromagnetic flowmeters have relatively high cost and their practical application in millimeter-scale pipes is also limited [3–5,10,11]. Therefore, it is necessary to seek more effective methods to solve the problem of flow rate measurement in millimeter-scale pipes.

Electrical conductivity is one of the basic physical parameters. Conductivity detection techniques have been adopted to implement flow rate measurements for many years and some achievements/progress have been obtained [12,13]. However, the conventional conductivity detection technique for flow rate measurement is based on contact conductivity detection. The detection electrodes are directly in contact with the fluid. That will cause polarization effects and electrochemical erosion effects. Meanwhile, if the electrode has been contaminated, unpredictable measurement errors will arise. Besides, the conventional conductivity detection techniques for flow rate measurement are used in normal scale pipes and few research works concerning the application of conductivity detection technique to the flow rate measurement in millimeter-scale pipes have been reported.

Capacitively Coupled Contactless Conductivity Detection (C^4^D) is a new conductivity detection technique, which was independently proposed by Zemann *et al.*, da Silva and do Lago, in 1998 [14,15], on the basis of Gas' research work [16]. Compared with the conventional contact conductivity detection technique, C^4^D is a contactless detection technique and the electrodes of a C^4^D sensor are not in contact with the measured fluid. C^4^D can avoid the electrochemical reactions on the electrode surfaces and the polarization of the electrodes. Meanwhile, C^4^D sensors are low cost and more robust [17–20]. Unfortunately, C^4^D is still a developing technique. To date, C^4^D has mainly been studied and applied in the research field of analytical chemistry for ion concentration/conductivity detection in capillaries (the inner diameter is usually less than 0.2 mm) [14–20]. Little research concerning the application of C^4^D to the flow rate measurement in millimeter-scale pipes has been reported [17–19,21–24].

The aim of this work was to propose a new method for flow rate measurement in millimeter-scale pipes, combining the C^4^D technique and the principle of cross correlation flow measurement. The research work included two steps: first, a new five-electrode C^4^D sensor, which was suitable for the flow rate measurement in millimeter-scale pipes, was developed. Second, with two conductivity signals obtained by the developed sensor, the flow rate measurement using the principle of cross correlation flow measurement was implemented. Meanwhile, experiments were carried out in five millimeter-scale pipes with different inner diameters of 0.5, 0.8, 1.8, 3.0 and 3.9 mm, respectively, to verify the feasibility and effectiveness of the proposed flow rate measurement method.

## 2. Five-Electrode C^4^D Sensor

**Figure 1.**
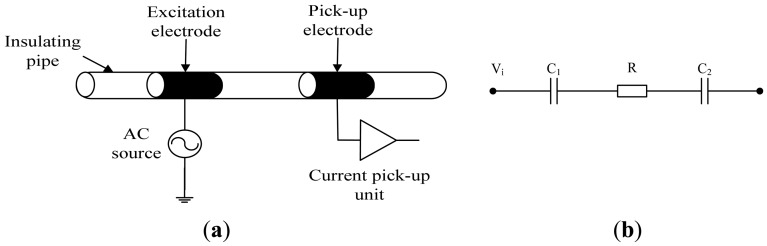
Principle of C^4^D technique. (**a**) Construction of conventional C^4^D sensor. (**b**) Simplified equivalent circuit of conventional C^4^D sensor.

Figure 1 inllustrates the principle of the C^4^D technique. As shown in Figure 1(a), a conventional C^4^D sensor includes an insulating pipe, two metal electrodes (an excitation electrode and a pick-up electrode) placed cylindrically around the outside of the insulating pipe, an AC source and a current pick-up unit. Figure 1(b) shows the simplified equivalent circuit of conventional C^4^D sensor. *C_1_* and *C_2_* are the coupling capacitances formed by the two metal electrodes, the insulating pipe and the conductive fluid. *R* is the equivalent resistor of the fluid between the two electrodes. Thus, an alternating current path is formed. The application of an AC voltage *V_i_* on the excitation electrode will lead to an AC current flowing through the AC path. From the AC current obtained by the AC current pick-up unit, the conductivity detection can be implemented [14–19].

For the conventional C^4^D sensor, from Figure 1 it can be seen that only the impedance of the resistor (*R*) is the useful signal. The impedances of the two coupling capacitances (*C_1_* and *C_2_*) are the unfavorable background signals. The existence of the background signals has a negative influence on the conductivity detection and hence limits the resolution and the detection range [18–27], so it is necessary to find an effective method to solve this problem [18–20]. Laugere *et al.* have proposed a new detection method and have developed a four-electrode C^4^D sensor [25–28]. In the four-electrode C^4^D sensor, four electrodes are placed cylindrically around the pipe. The outer two are excitation electrodes and the inner two are pick-up electrodes. A fixed AC current source is connected between the outer electrodes and a resulting differential voltage between two inner electrodes can be obtained by a high input impedance voltmeter. Thus, from the measured differential voltage, the measurement value of the fluid conductivity can be obtained. Compared with the conventional C^4^D sensor, the way of implementing the conductivity detection is different. The conventional current measurement way has been changed to a voltage measurement way, thus the existence of coupling capacitances (*C_1_* and *C_2_*) has no influence on the conductivity measurement. Unfortunately, the detection method proposed by Laugere *et al.* is also a developmental technique. According to the latest technique reports, the four-electrode C^4^D sensor has been used in a channel with dimensions of 106 μm × 170 μm [24–27]. However, there is still a lack of the knowledge and experience of the four-electrode C^4^D sensor in millimeter-scale pipes.

**Figure 2.**
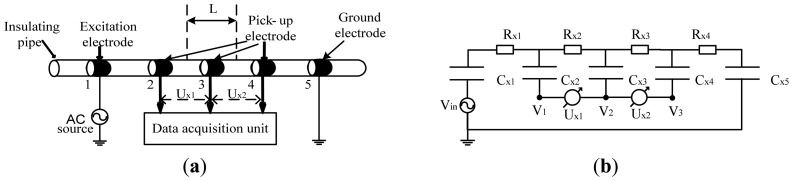
The new five-electrode C^4^D sensor. (**a**) The construction of five-electrode C^4^D sensor; (**b**) The simplified equivalent circuit.

Although more research work should be undertaken, the detection method proposed by Laugere *et al.* provided a very useful reference for our research work. In this work, on the basis of Laugere *et al.*'s work, we developed a new five-electrode C^4^D sensor in millimeter-scale pipes. The construction of the new five-electrode C^4^D sensor was illustrated in Figure 2(a), along with a simplified equivalent circuit diagram given in Figure 2(b). The aim of this work was to implement the flow rate measurement using the principle of cross correlation flow measurement. According to the principle of cross correlation flow measurement, two independent conductivity signals were needed. A four-electrode C^4^D sensor could only obtain one conductivity signal, so an extra electrode was added. The new sensor became a five-electrode C^4^D sensor.

The new five-electrode C^4^D sensor consists of an insulating pipe, five cylindrical metal electrodes (one excitation electrode, one ground electrode and three pick-up electrodes), an AC source and a data acquisition unit. *C_x1_∼C_x5_* are the coupling capacitances formed by the five metal electrodes, the insulating pipe and the conductive fluid. *R_x1_∼R_x4_* are the equivalent resistors of the fluid between the two adjacent electrodes. *C_x1_*, *R_x1_∼R_x4_* and *C_x5_* form an alternating current path. When an AC voltage *V_in_* is applied on the excitation electrode, an AC current *I* flowing through the AC path is generated. The AC current *I* can be determined by:

(1) $$I=\frac{V_{in}}{Z}$$

where *V_in_* is the input voltage (AC source), and *Z* is the overall impedance of the AC path. At the frequency *f* of the input voltage signal, the overall impedance *Z* is:

(2) $$Z=R_{x1}+R_{x2}+R_{x3}+R_{x4}‐j\left(\frac{1}{2\pi fC_{x1}}+\frac{1}{2\pi fC_{x5}}\right)$$

The differential voltage *U_x1_* reflects the conductivity information of the fluid between electrode 2 and electrode 3 (up-stream sensor) and the differential voltage *U_x2_* reflects the conductivity information of the fluid between electrode 3 and electrode 4 (down-stream sensor). *U_x1_* and *U_x2_* can be expressed as:

(3) $$U_{x1}=IR_{x2}$$

(4) $$U_{x2}=IR_{x3}$$

Thus, two conductivity signals can be obtained by measuring the differential voltages *U_x1_* and *U_x2_*.

Comparing Figures 1 and 2, it can be seen that the difference between the conventional C^4^D sensor and the new five-electrode C^4^D sensor is the way conductivity detection is implemented. The new five-electrode C^4^D sensor is based on the detection method proposed by Laugere *et al.*, in which the conductivity measurement is mainly implemented by the voltage measurement technique and the existence of coupling capacitances has no influence on the conductivity measurement. Thus, in the new five-electrode C^4^D sensor, the unfavorable influence of the coupling capacitances on conductivity detection can be avoided and its linearity, accuracy, and sensitivity of the measurement can be improved [18,25–27]. However, with the increasing dimension of the pipes, the impedances of the fluid resistors *R_x2_* and *R_x3_* decrease. That causes more difficulties for the low voltage measurement and electrical circuit design in millimeter-scale pipes. The electrical circuit and the signal processing circuit need to be specially designed in our research work.

The data acquisition unit can be roughly divided into two parts: the signal processing circuit and the data processing computer system. The function of the signal processing circuit is to demodulate and to amplify the conductivity signals obtained from the AC path. The function of the data processing computer system is to acquire the data and display the detection results (in this work, the data processing computer system was based on the data acquisition module of National Instruments (NI cDAQ-9172) and a microcomputer).

**Figure 3.**
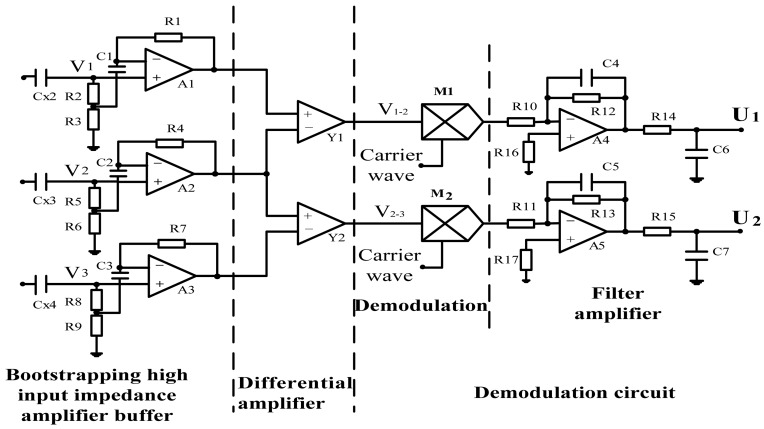
The signal processing circuit.

As shown in Figure 3, the signal processing circuit includes three parts: (1) Bootstrapping high input impedance amplifier buffer, which is used to obtain the voltage signals *V_1_*, *V_2_*, *V_3_*. (2) Differential amplifier, which is used to obtain two differential voltage signals *V_1-2_*, *V_2-3_*. (3) Demodulation circuit, which consists of Demodulation and Filter amplifier. Finally, two independent conductivity signals of fluid *U_1_*, *U_2_* are obtained. Using these two independent conductivity signals, the flow rate measurement can be implemented according to the principle of cross-correlation measurement [4,5,29–31].

## 3. Flow Rate Measurement Implementation

The new five-electrode C^4^D sensor can obtain two independent conductivity signals (the differential voltages *U_1_* and *U_2_*). According to the principle of cross correlation flow measurement, if we cross-correlate the two conductivity signals, the time delay τ between the two signals can be determined by searching for the time position of the maximum cross-correlation coefficient. The cross-correlation function *R*_*U*_1___*U*_2__(*τ*) can be defined as:

(5) $$R_{U_{1}U_{2}}(\tau )=\underset{T\to \infty }{\lim}\frac{1}{T}\int_{0}^{T}U_{1}(t)U_{2}(t+\tau )dt$$

The cross-correlation coefficient *ρ*_*U*_1___*U*_2__(*τ*) can be defined as:

(6) $$\rho_{U_{1}U_{2}}(\tau )=\frac{R_{U_{1}U_{2}}(\tau )}{\sqrt{R_{U_{1}U_{1}}(0)R_{U_{2}U_{2}}(0)}}$$

Then the average flow velocity *V* can be calculated by the following equation [4,5,29–31]:

(7) $$V=\frac{L}{\tau }$$

where *L* is the distance between the centre of the combination of electrode 2 and electrode 3 and the centre of the combination of electrode 3 and electrode 4, as shown in Figure 2 (in the developed five-electrode C^4^D sensor, the combination of electrode 2 and electrode 3 and the combination of electrode 3 and electrode 4 can be regarded as two independent sensors of conductivity detection, the up-stream sensor and the down-stream sensor).

Thus, the flow rate *Q_0_* can be calculated by the following equation:

(8) $$Q_{0}=AV$$

where *A* is the sectional area of the pipe. Meanwhile, for practical cross correlation flow rate measurement, calibration is needed. The practical flow rate *Q* should be:

(9) Q=KAV

where *K* is the calibration coefficient.

## 4. Experimental Results and Discussion

The experiments included two parts: first, the conductivity measurement experiments of the new five-electrode C^4^D sensor in millimeter-scale pipes were carried out to test the conductivity measurement performance of the new five-electrode C^4^D sensor. Second, the flow rate measurement experiments were carried out to verify the effectiveness of the proposed flow rate measurement method in millimeter-scale pipes.

### 4.1. The Conductivity Measurement Experiments

The conductivity measurement experiments of the new five-electrode C^4^D sensor were carried out in five millimeter-scale pipes with different inner diameters of 0.5, 0.8, 1.8, 3.0 and 3.9 mm, respectively. The comparison conductivity measurement experiments between the new five-electrode C^4^D sensor and the conventional C^4^D sensor were also carried out. A commercial contact conductivity meter (FE30, Mettler Toledo Inc., 0.00 μS/cm∼199.9 mS/cm, ±0.5%F.S.) was used to obtain the reference conductivity data. The AC source was a function generator (CA1640-02, RIGOL Technologies Inc.). The signal frequency of the input voltage (AC source) was 10 kHz for the new five-electrode C^4^D sensors with the inner diameter of 0.5 and 0.8 mm, 60 kHz for the new five-electrode C^4^D sensors with the inner diameter of 1.8 and 3.0 mm and 120 kHz for the new five-electrode C^4^D sensors with the inner diameter of 3.9 mm. The experimental material was KCl solution. The conductivity range of KCl solution was 0.1865 mS/cm∼13.21 mS/cm. The temperature was around 26 °C. The electrodes were made by painting five rings of silver paint over the glass pipes. The length of the electrodes and the distance between two adjacent electrodes were all 10 mm. The output of the new five-electrode C^4^D sensors was volt signal and the noise was millivolt signal. The signal‐to‐noise‐ratio could meet the requirement of the measurement. The output signals (the differential voltages U_1_ and U_2_) reflected the conductivity of KCl solutions, which could be seen from the Equations (3) and (4). Two calibration curves, which reflected the relationship between the conductivity of KCl solutions and the differential voltages U_1_, U_2_, were pre-determined respectively by experiments. Then the measurement values of conductivity were determined.

Relative difference between the reference data obtained by the commercial contact conductivity meter and the measurement value obtained by the new five-electrode C^4^D sensor was used to analyze the conductivity measurement results of the new five-electrode C^4^D sensor. The relative difference *d_c_* can be calculated by the following equation:

(10) $$d_{c}=\frac{S_{m}-S_{r}}{S_{r}}\times 100\%$$

where *S_r_* is the reference data obtained by the commercial contact conductivity meter, and *S_m_* is the measurement value obtained by the new five-electrode C^4^D sensor.

**Figure 4.**
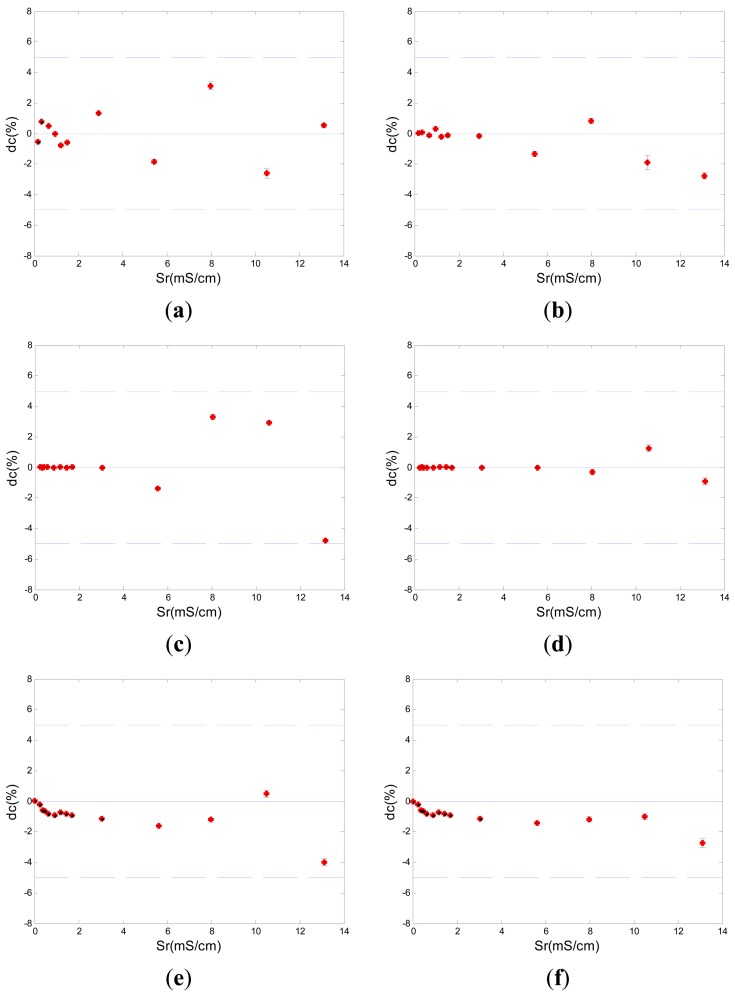

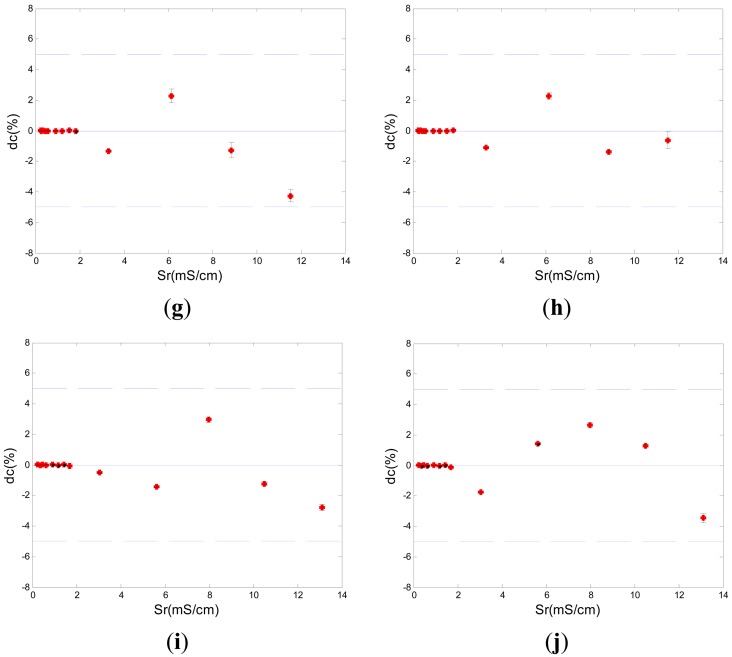
Experimental results by using the new five-electrode C^4^D sensor in five pipes. (**a**) the up-stream sensor in 0.5 mm i.d. pipe. (**b**) the down-stream sensor in 0.5 mm i.d. pipe. (**c**) the up-stream sensor in 0.8 mm i.d. pipe. (**d**) the down-stream sensor in 0.8 mm i.d. pipe. (**e**) the up-stream sensor in 1.8 mm i.d. pipe. (**f**) the down-stream sensor in 1.8 mm i.d. pipe. (**g**) the up-stream sensor in 3.0 mm i.d. pipe. (**h**) the down-stream sensor in 3.0 mm i.d. pipe. (**i**) the up-stream sensor in 3.9 mm i.d. pipe. (**j**) the down-stream sensor in 3.9 mm i.d. pipe (S_r_ is the reference data obtained by the commercial contact conductivity meter. *d_c_* is the relative difference between the reference data obtained by the commercial contact conductivity meter and the measurement data obtained by the new five-electrode C^4^D sensor. The upper and lower lines are defined as *d_c_* = ±5%).

**Figure 5.**
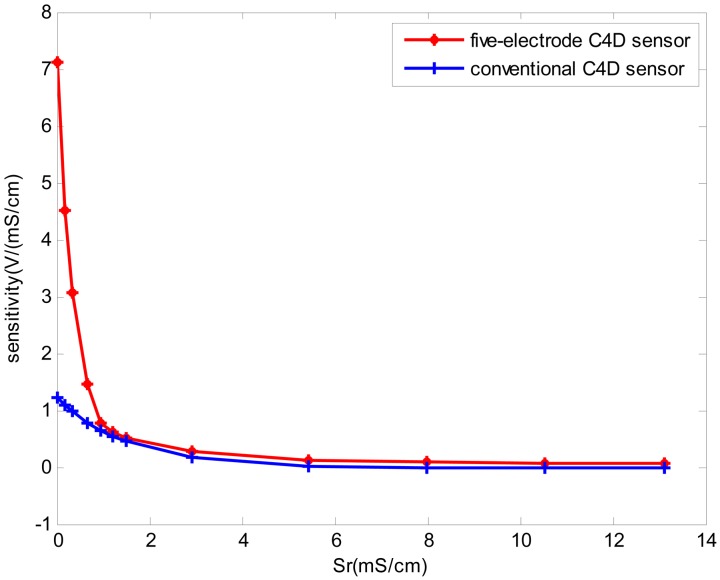
A typical sensitivity plot of the experiments in 0.5 mm i.d. pipe.

Figure 4 showed a typical group of experimental results of five new five-electrode C^4^D sensors in millimeter-scale pipes with different diameters. Figure 5 showed a typical sensitivity plot of the new developed five-electrode C^4^D sensor (the inner diameter of the pipe was 0.5 mm) and the conventional C^4^D sensor (the inner diameter of the pipe was also 0.5 mm). The sensitivity can be defined as Δ*U*/Δ*S* (*U* is the voltage obtained by the sensor, and *S* is the value of conductivity). The experimental results showed that the design of the new five-electrode C^4^D sensor was successful. The measurement accuracy of the new five-electrode C^4^D sensor was satisfactory. Compared with the commercial contact conductivity meter, the maximum relative differences *d_c_* between the reference data obtained by the commercial contact conductivity meter and the measurement values obtained by the five new five-electrode C^4^D sensors (either obtained by the up-stream sensor or obtained by the down-stream sensor) were all less than 5%. The conductivity measurement experiments verified the new five-electrode C^4^D sensor was suitable for the cross correlation flow rate measurements in millimeter-scale pipes. Meanwhile, the experimental results also showed that the detection method proposed by Laugere *et al.* was suitable for the conductivity measurement in millimeter-scale pipes. That provided a useful reference/experience for other researchers' work.

### 4.2. The Flow Rate Measurement Experiments

**Figure 6.**
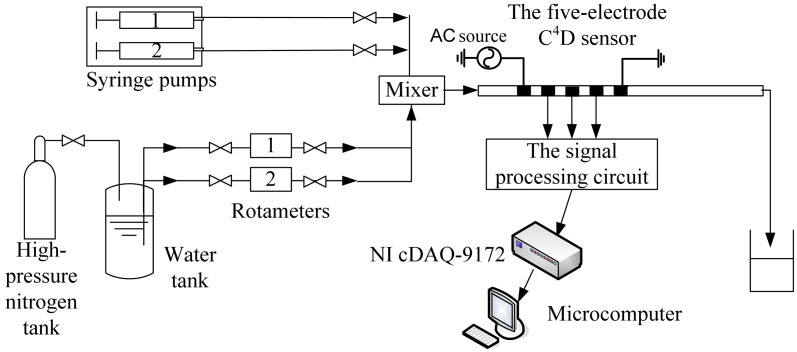
Experimental setup.

Figure 6 illustrated the experimental setup for flow rate measurement in millimeter-scale pipes. Tap water was the experimental material and milk acted as the tracer (since the stochastic fluctuation signals in single-phase flow is quite weak [29–31], cross correlation flow measurement systems for single-phase flow usually use tracers in the pipe to effectuate stronger stochastic fluctuation signals and better signal-to-noise ratios [12,30,31]. In this work, milk was used as the tracer and it was slowly injected by a syringe pump). Tap water was driven into the pipe by syringe pump 1 or the high-pressure nitrogen tank. Milk was driven into the pipe by syringe pump 2. Tap water and milk were mixed at the mixer, flowed through a horizontal pipe and then passed through the new five-electrode C^4^D sensor. If the fluid flow rate was less than 3.6 L/h (60 mL/min), tap water was driven by syringe pump 1. The reference flow rate was obtained by syringe pump 1 (flow rate range: 0∼60 mL/min, accuracy: 0.35%). If the fluid flow rate ranged from 3.6 L/h to 25 L/h, tap water was driven by the high-pressure nitrogen tank. The reference flow rate was obtained by rotameter 1 (flow rate range: 2.5∼25 L/h, accuracy: 2.5%). If the fluid flow rate ranged from 25 L/h to 100 L/h, tap water was also driven by the high-pressure nitrogen tank. The reference flow rate was obtained by rotameter 2 (flow rate range: 25∼250 L/h, accuracy: 2.5%). The flow rate of milk tracer ranged from 0.006 L/h to 0.18 L/h.

The relative difference *d_f_*, which was used to analyze the flow rate measurement results of the new five-electrode C^4^D sensor, can be calculated by the following equation:

(11) $$d_{f}=\frac{Q_{m}-Q_{r}}{Q_{r}}\times 100\%$$

where *Q_r_* is the reference flow rate obtained by the rotameters or the syringe pumps, and *Q_m_* is the measured flow rate obtained by the new five-electrode C^4^D sensor.

**Figure 7.**
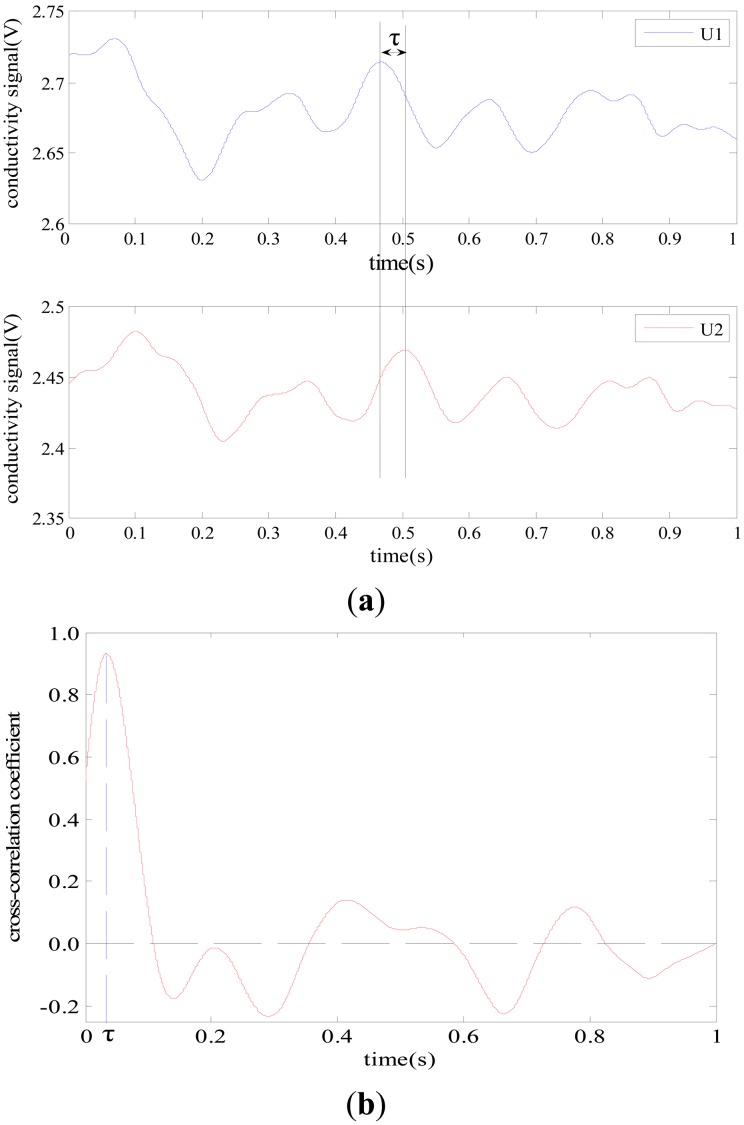
An example of the flow rate measurement. (**a**) Conductivity signals; (**b**) Cross-correlation coefficient curve.

According to the experimental results, the calibration coefficient *K* was 1.03 for the five-electrode C^4^D sensor with the inner diameter of 0.8 mm and was 1.0 for the five-electrode C^4^D sensor with the other four inner diameters, respectively. Figure 7 illustrates an example of the flow rate measurement. Figure 7(a) shows two conductivity signals obtained by the new five-electrode C^4^D sensor. Figure 7(b) shows the corresponding cross-correlation coefficient curve. The time delay between two conductivity signals *τ* = 0.0342 s, which was determined by seeking the peak position of the cross-correlation coefficient curve (Figure 7(b)). The maximum value of the cross correlation coefficient was 0.9322. In this work, the distance *L* = 20.0 mm, and the inner diameters of the glass pipe was 3.9 mm. Thus, the average flow velocity can be calculated, *v* = 0.5848 m/s, the flow rate *Q_m_* = 25.15 L/h. Meanwhile, the reference flow rate of tap water was obtained by the rotameter 1. In this experiment, the flow rate of tap water was 25 L/h and the flow rate of milk was 0.12 L/h. Thus, the reference flow rate *Q*_r_ = 25.12 L/h. Comparing *Q_m_* with *Q*_r_, it could be found that the relative difference *d_f_* of the flow rate was −0.12%.

**Figure 8.**
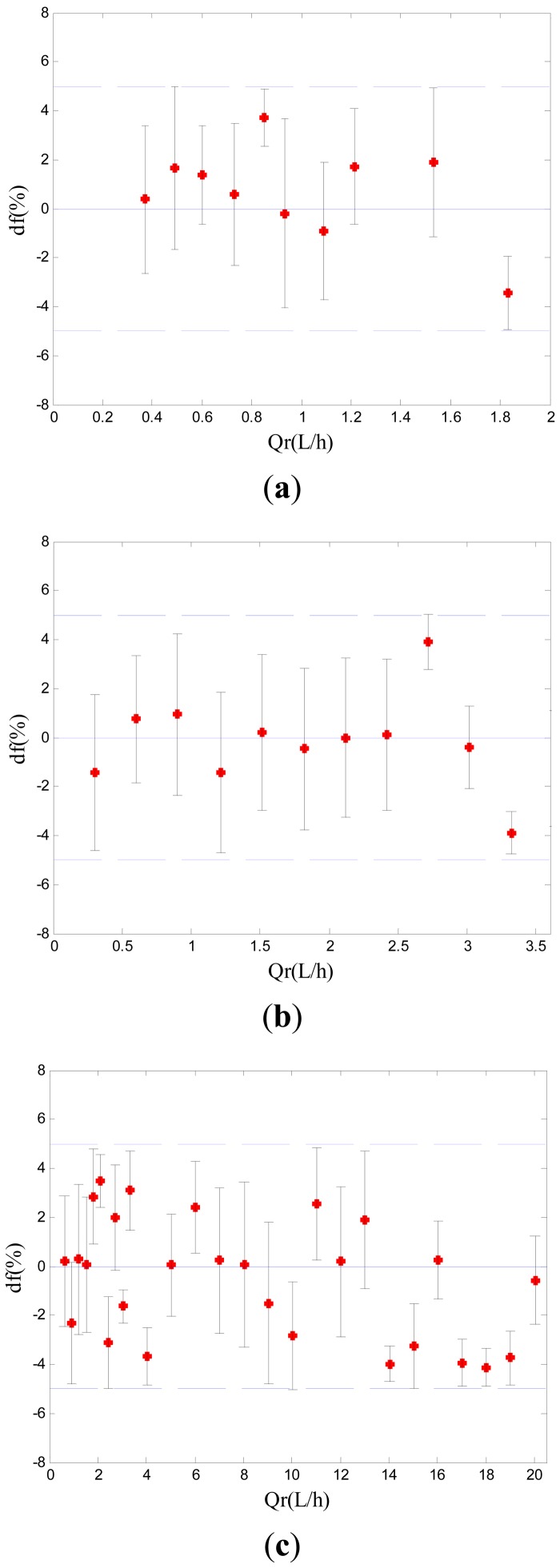

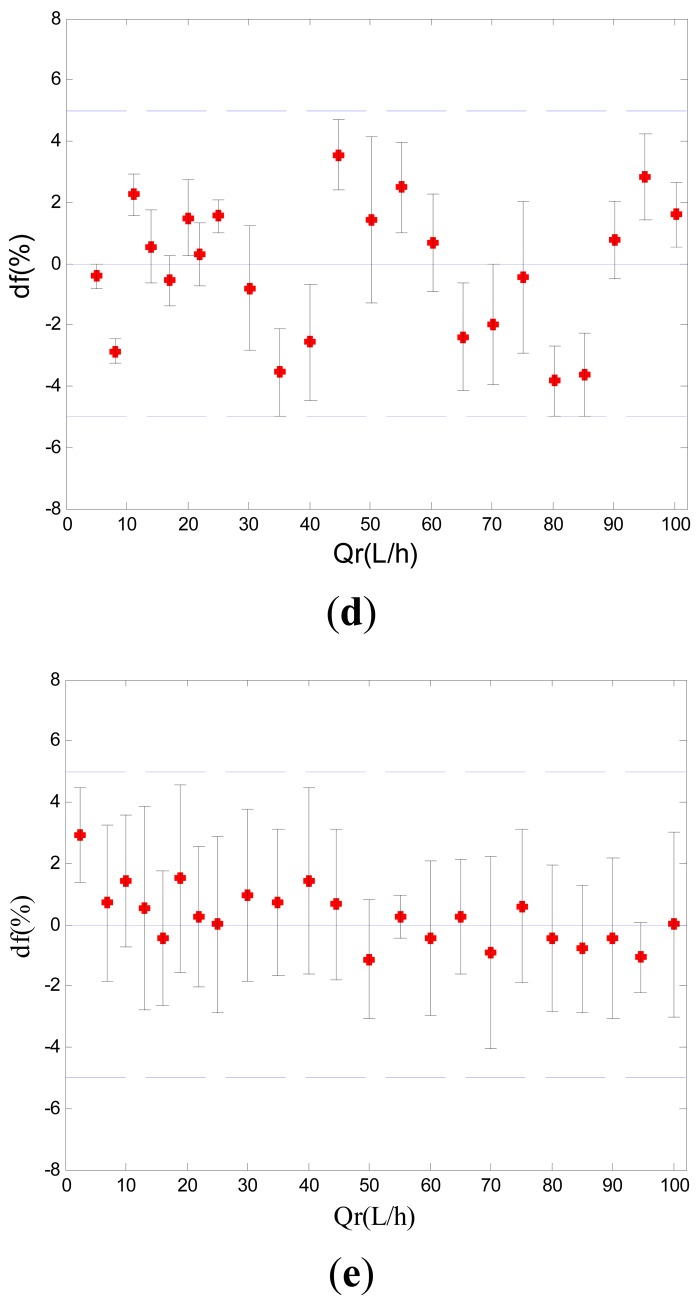
The experimental results in five millimeter-scale pipes. (**a**) Experimental results in 0.5 mm i.d. pipe. (**b**) Experimental results in 0.8 mm i.d. pipe. (**c**) Experimental results in 1.8 mm i.d. pipe. (**d**) Experimental results in 3.0 mm i.d. pipe. (**e**) Experimental results in 3.9 mm i.d. pipe (*Q_r_* is the reference flow rate obtained by the rotameters or the syringe pumps. d*_f_* is the relative difference between the reference flow rate obtained by the rotameters or the syringe pumps and the measured flow rate obtained by the new five-electrode C^4^D sensor. The upper and lower lines are defined as *d_f_* = ±5%).

Figure 8 showed the experimental results in five pipes with different inner diameters. The maximum relative difference *d_f_* between the reference flow rate and the measured flow rate was less than 5%. The experimental results showed that the proposed flow rate measurement method, which combined C^4^D technique and the principle of cross correlation flow measurement, was effective. The new five-electrode C^4^D sensor was successful and was suitable for the flow rate measurement in millimeter-scale pipes.

Compared with the commercial flowmeters (such as the constriction flowmeter, rotameter, turbine flowmeter, electromagnetic flowmeter and coriollis flowmeter, *etc.*) mentioned in Section 1, the proposed method in this work had advantages of low cost and contactless detection. Compared with the existing cross correlation flowmeters based on the conventional contact conductivity detection technique, the proposed method in this work could avoid the polarization of the electrodes and the electrochemical erosion on the electrode surfaces. Furthermore, the proposed method was suitable for the flow rate measurement in millimeter-scale pipes.

## 5. Conclusions

In this research, a new method, which combined the C^4^D technique and the principle of cross correlation flow measurement, was proposed for flow rate measurement in millimeter-scale pipes. A new five-electrode C^4^D sensor was developed. Meanwhile, experiments were carried out in five millimeter-scale pipes with different inner diameters. The research results showed that the flow rate measurement of this method had a relative difference lower than 5%, which was considered accurate and satisfactory for the flow rate measurement application in millimeter-scale pipes. As a preliminary study, this research work verified the feasibility of the application of C^4^D technique to the flow rate measurement in millimeter-scale pipes. However, more research works (improving the measurement accuracy, extending the application scope, *etc.*) should be undertaken in this area. Optimization of the length of the electrodes and the distance between two adjacent electrodes will be topics for our further research works. Meanwhile, to improve the signal-to-noise rate of the five-electrode C^4^D sensor and then to implement the flow rate measurement without the help of the tracer is also a challenge in future.
